# Catalyst free one pot three components synthesis of 2-iminothiazoles from nitroepoxides and thiourea

**DOI:** 10.1038/s41598-023-30243-5

**Published:** 2023-02-22

**Authors:** Elham Badali, Azim Ziyaei Halimehjani, Azizollah Habibi

**Affiliations:** 1 Faculty of Chemistry, Kharazmi University, 49 Mofateh St., P. O. Box 15719-14911, Tehran, Iran; 2 Department of Chemistry, Sharif University of Technology, P. O. Box 11155-9516, Tehran, Iran

**Keywords:** Chemistry, Organic chemistry, Synthetic chemistry methodology

## Abstract

Nitroepoxides were introduced as efficient substrates for the one-pot three-component synthesis of 2-iminothiazoles under catalyst-free conditions. Reaction of amines, isothiocyanates, and nitroepoxides in THF at 10–15 °C afforded corresponding 2-iminothiazoles in high to excellent yields. The reaction proceeds via the in situ formation of thiourea from an amine and an isothiocyanate, followed by nitroepoxide ring opening with the sulfur of thiourea, cyclization reaction, and dehydration cascade. The structures of products were confirmed by IR, NMR, HRMS analyses and X-ray crystallography.

## Introduction

**Figure 1:**
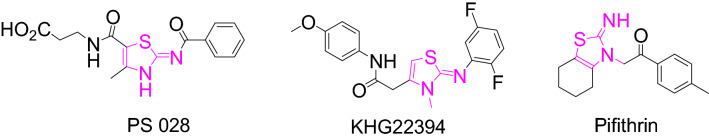
Biologically active 2-imino-1,3-thiazolines.

Nitrogen and sulfur containing heterocyclic compounds such as thiazoles are of great practical and fundamental interest. Development of novel synthetic strategies for the synthesis of thiazole derivatives with various substitution pattern have received considerable attention in the field of pharmaceutical and biomedical chemistry^1–5^. Particularly, the 2-imino-1,3-thiazolines are available in many biologically active compounds with antimicrobial, anticancer, anti-inflammatory, antihistaminic, antihypertensive, hypnotic, and anticonvulsant activities^6–13^. In addition, they were applied for the identification of human cells with positive myeloperoxidase reactivity^14^. For example 4-methyl-3-*H*-thiazoline derivative, PS-028, is a potent and selective GPIIb/IIIa receptor antagonist (Fig. 1)^15,16^. Also, KHG22394 containing 2-imino-1,3-thiazoline moiety is known as a melanin production inhibitor in a dose-dependent manner while not directly inhibit tyrosinase as a rate-limiting melanogenic enzyme and also applied for the production of skin whitening cream (Fig. 1)^17^. Another example with thiazoline core is Pifithrin, which is widely used as a p53 transcription inhibitor in cells and could restrict cellular apoptosis (Fig. 1)^18,19^. Furthermore, thiazolines have been found applications as acaricides, insecticides, and plant growth regulators in agriculture^20,21^.

Nitroepoxides, which can be simply prepared from nitroalkenes by epoxidation reaction with H_2_O_2_/NaOH^22^, are efficient intermediates in synthetic organic chemistry and were applied extensively as synthons of vicinal double electrophilic compounds such as *α*-diones and *α*-haloketones for the synthesis of valuable heterocyclic compounds^23^. Although the synthesis of nitroepoxides and their ring opening with heteroatom-centered nucleophiles such as sodium phenoxides, sodium thiophenolates, and amines came back to around 50 years ago^24^, but research on these compounds was accelerated in recent 15 years. Diversities of mono nucleophiles such as potassium xanthates, dithiocarbamic acids, sodium aryl sulfonates and sodium bisulfite were added to nitroepoxides to obtain the corresponding *α*-heterosubstituted ketones^23,25^. In addition, *bis*-nucleophiles such as *N*-alkyl(thio)ethanolamine^26^, amidines^27^, diamines^28^, *S*-alkyl dithiocarbamates^23^ and etc.^29–31^ have been utilized in the reaction with nitroepoxides for the synthesis of valuable heterocyclic compounds including imidazoles, 1,3-thiazoles, benzodiazepines, thiazoline-2-thiones, and pyrazines.

**Scheme 1:**
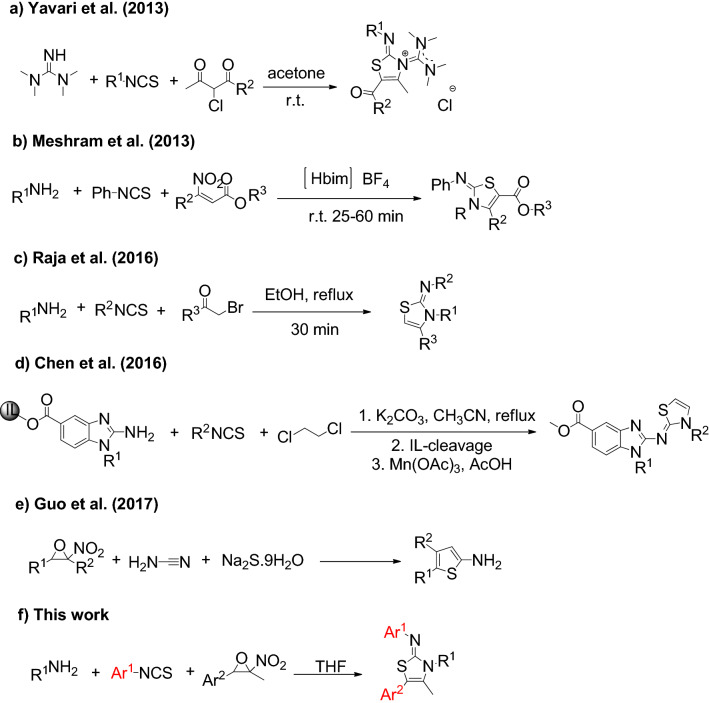
Reported and proposed MCRs for the synthesis of 2-imino-1,3-thiazolines.

Multicomponent reactions (MCRs) provide simple access to complex molecules in a one-pot. MCRs have several advantages such as generation of several bonds in a single operation, avoiding the need for isolation and purification of reaction intermediates, saving in time, materials, solvents and energy^32–38^. For this purpose, multicomponent reaction of isothiocyanates with amines in the presence of an electrophile is an efficient strategy for the synthesis of 2-imino-1,3-thiazolines. In this context, Yavari et al. developed a synthetic route for the synthesis of functionalized 2-imino-1,3-thiazoles using tetramethylguanidine, isothiocyanates, and 2-chloro-1,3-dicarbonyls (Scheme 1a)^39^. Meshram and co-workers investigated the one-pot three-component reaction of amines, phenyl isothiocyanates and *β*-nitroacrylates in [Hbim]BF_4_ ionic liquid (Scheme 1b)^40^. Furthermore, Raja et al. reported the reaction of aryl isothiocyanates, phenacyl bromides, and aryl amines in ethanol as green reaction media in 2016 (Scheme 1c)^41^. Chen and coworkers described the synthesis of 2-imino-1,3-thiazolidines and 2-imino-1,3-thiazolines through the reaction of ionic liquid attached 2-aminobenzimidazoles with isothiocyanates and 1,2-dichloroethane, followed by ionic cleavage with methanolysis reaction and oxidation with Mn(OAc)_3_ (Scheme 1d)^42^. In 2017, a one-pot three-component route for the synthesis of 2-aminothiazoles was developed by Guo et al*.* via the reaction of nitroepoxides with cyanamide and sodium sulfide. The scope of this reaction is limited to the use of nitroepoxides and other components cannot be changed (Scheme 1e)^43^. Beside multicomponent reactions, condensation reaction of *N,Nʹ*–disubstituted thiourea with *α*-haloketones and ring transformation of 2-(thiocyanomethyl) aziridines were also developed for the synthesis of 2-imino-1,3-thiazolines^44–47^.

## Experimental section

### General

All chemicals and solvents were obtained from commercial sources and used as received. The ^1^H and ^13^C NMR spectra of products were recorded on a Bruker AMX 300 MHz spectrometers referenced to internal Me_4_Si at 0.00 ppm. Reaction monitoring was carried out by thin-layer chromatography using TLC silica gel 60 F254 plates. HRMS (High Resolution Mass Spectra) was measured on a THERMO SCIENTIFIC Advantage and a THERMO SCIENTIFIC Exactive instrument equipped with an APCI source in the positive-ion mode. Mass analysis was performed using Agilent Technology (HP); 5973 Network Mass Selective Detector equipped with EI mode and ionization energy of 70 eV. The temperatures of the electronic-impact ion source and MS quadrupole were 230 °C. IR spectra were recorded on a Perkin-Elmer Spectrum RXI FT-IR Spectrometer. Nitroepoxides were prepared according to the literature procedures^22,48^.

### General procedure for the synthesis of 4a-s

In a test tube equipped with magnetic stirrer bar, an isothiocyanate (1.0 mmol, 1 equiv), an amine (1.2 equiv) and THF (4 mL) were mixed for 1 h at room temperature. Progress of the reaction was monitored by TLC. After complete consumption of the isothiocyanate, the reaction temperature was decreased to 10–15 °C and a nitroepoxide (1 equiv) was added and the mixture was stirred at 10–15 °C for 6 h. The solvent was removed under reduced pressure to afford yellow viscous oil. Purification was carried out by recrystallization in a minimum amount of MeOH or by column chromatography using silica gel and *n*-hexane/EtOAc (7/3) (for compounds **4f.**, **4 h**, and **4i**). The structures of products were confirmed by IR, ^1^H NMR, ^13^C NMR, Mass/HRMS analyses and X-ray crystallography. Detailed information is available in the “Supporting Information” file.

## Results and discussion

In continuation of our interest on the synthesis of *N,S*-heterocycles and the chemistry of nitroepoxides^49–52^, herein we report a novel catalyst-free one-pot three-component route for the synthesis of 2-imino-1,3-thiazolines via nitroepoxide ring opening reaction (Scheme 1f). Reaction of ethylamine **1** with phenyl isothiocyanate **2** and nitroepoxide **3a** was considered as a model reaction for optimization of the reaction conditions. Mixing an equimolar amount of ethylamine and **2** in a solvent such as water or DMF for 1 h at room temperature, followed by the addition of nitroepoxide (1 equivalent) and further stirring for 6 h at room temperature afforded a trace amount of corresponding product **4a** (Table 1, entries 1 and 3). By decreasing the reaction temperature to 10–15 °C for the second step, the yield was improved to 25% and 18% in water and DMF, respectively (Table 1, entries 2 and 4). By performing the reaction in CH_2_Cl_2_ at the same temperature, the yield was improved to 55% (Table 1, entry 5). Using ethanol as reaction solvent increased the yield to 70% (Table 1, entry 6). In addition, using THF as solvent improved the yield to 80% at 10–15 °C for the total time of 7 h (Table 1, entry 7). Interestingly, by using 1.2 equivalent of amine, the yield was improved to 89% (Table 1, entry 8). Prolonging the reaction time to 10 h and overnight didn’t improve the reaction yield (Table 1, entries 9 and 10). It is notable that premixing of ethylamine and phenyl isothiocyanate for 1 h at room temperature before the addition of nitroepoxide is crucial to obtain high yield. Finally, reaction of 1.2 equivalent of an amine with an equivalent of isothiocyanate in THF for 1 h at room temperature, followed by the addition of a nitroepoxide (1 equiv.) and further stirring for 6 h at 10–15 °C was considered as optimal reaction conditions for further derivatization.

**Table 1:** Optimization of the reaction conditions.


Entry	**2a** (equiv)	Solvent	*T* (°C)	*t* (h)	Yield^a^ (%)
1	1	H_2_O	rt	6	Trace
2	1	H_2_O	10–15	6	25
3	1	DMF	rt	6	Trace
4	1	DMF	10–15	6	18
5	1	DCM	10–15	6	55
6	1	EtOH	10–15	6	70
7	1	THF	10–15	6	80
**8**	**1.2**	**THF**	**10–15**	**6**	**89**
9	1.2	THF	10–15	10	85
10	1.2	THF	10–15	24	85

^a^Isolated yield.

**Figure 2:**
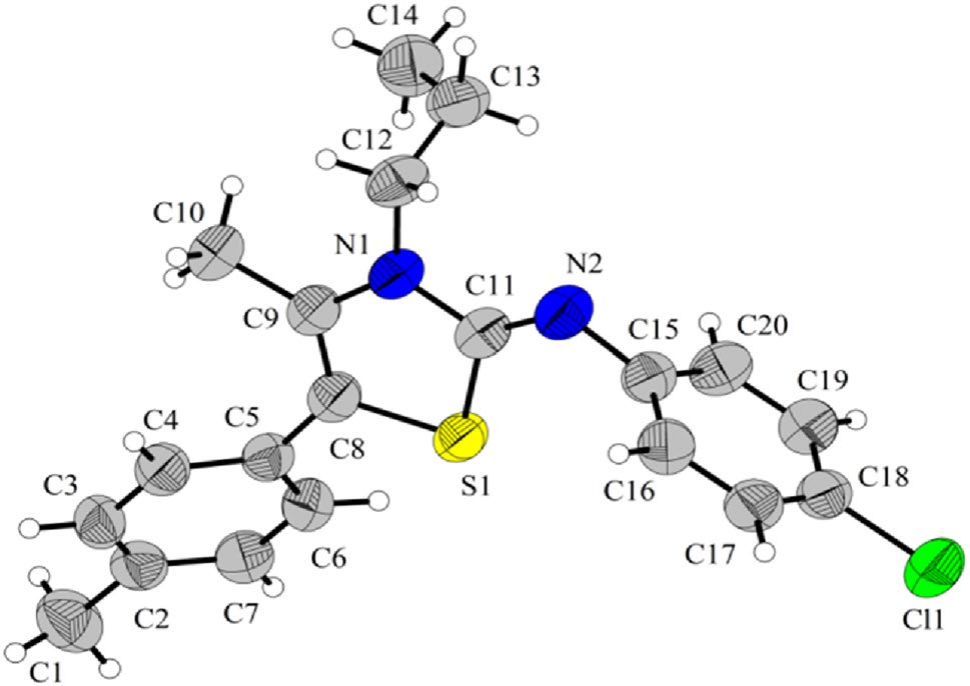
ORTEP representation of the structure **4p**. The figure was drawn by DIAMOND (https://www.crystalimpact.com/diamond/)^53^.

With optimized reaction conditions in hand, the generality and scope of this protocol was examined using various amines, isothiocyanates, and nitroepoxides and the results are summarized in Table 2. Diversities of primary aliphatic amines including ethylamine, propylamine, buthylamine, isobuthylamine, benzylamine were applied successfully in this protocol. All aliphatic primary amines afforded high to excellent yields in this protocol. In contrast to aliphatic amines, aromatic amines were not compatible with this protocol. Slightly higher yields were obtained with isothiocyanates with an electron-withdrawing group on the phenyl ring compared to simple phenyl isothiocyanate (for example **4r** vs. **4 g**). In addition, the nature and position of the substituents on the phenyl ring of nitroepoxides did not have significant impact on the reaction yield. High to excellent yields were obtained with both electron-donating and -withdrawing substituents on the phenyl ring. The structures of products were confirmed using IR, ^1^H and ^13^C NMR, and HRMS analyses. Characteristic signal in ^1^H NMR spectra is related to the methyl group which appeared around 2.2 ppm in all products. In addition, a signal at 158 ppm in ^13^C NMR was assigned for the carbon of the imine moiety in the products. In principle, two regioisomers may be generated in the reaction of in situ prepared thiourea with nitroepoxide, which is difficult to differentiate by ^1^H and ^13^C NMR analyses. For this purpose, to confirm the proposed structure for the products, the structure of **4p** was elucidated using single-crystal X-ray analysis. ORTEP representations of **4p** is shown in Fig. 2 (CCDC no. 217830; for details of the crystal structure data and refinement of 4p see the Supporting Information). X-ray analysis revealed that the configuration around the imine bond is *Z*, in which the substituent on the nitrogen of imine is in the same side with sulfur atom in the ring.

**Table 2:**
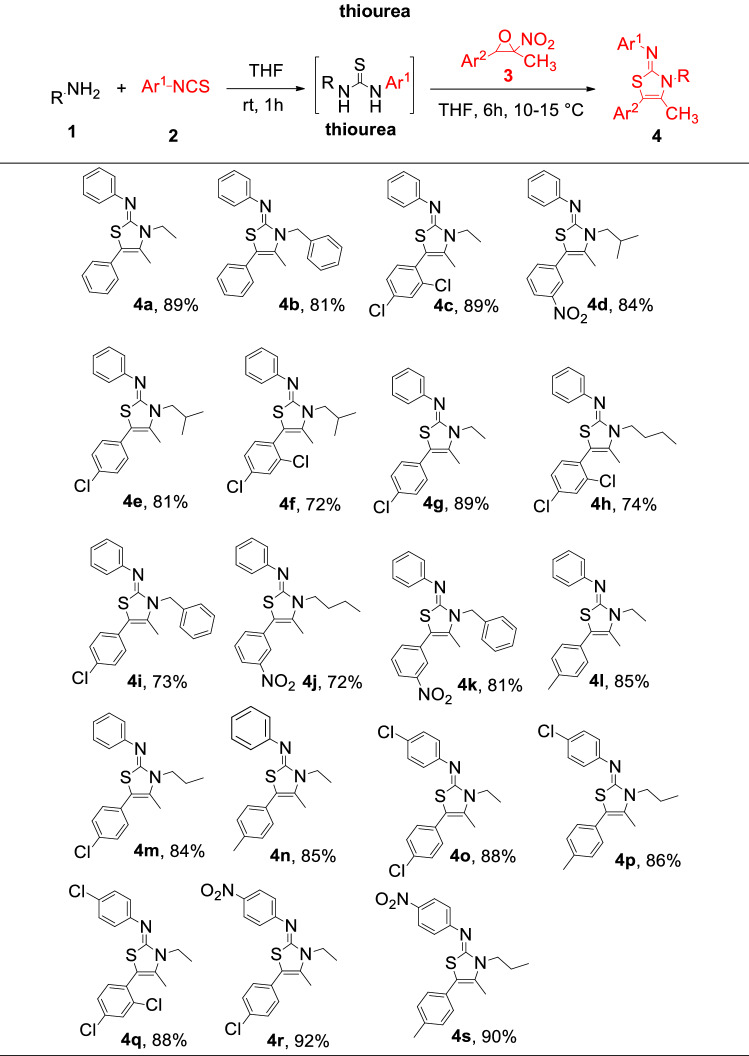
Diversity in the synthesis of 2-iminothiazoles^a,b^.

^a^Reaction conditions: amine (1.2 equiv) and arylisothiocyanate (1 equiv) in THF for 1 h, then nitroepoxide (1 equiv), THF, 10–15 °C for 6 h.

^b^Isolated yield.

**Scheme 2:**
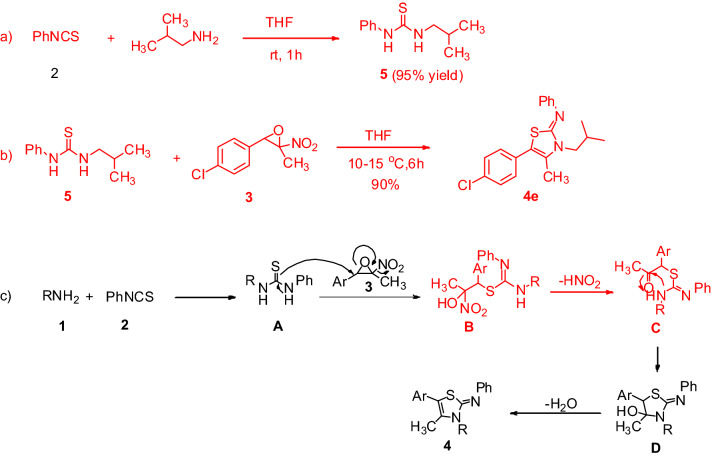
Control experiments (**a**,**b**) and proposed mechanism for the formation of 2-imino-1,3-thiazoline (**c**).

To propose a reasonable mechanism, two control experiments were carried out. Reaction of phenylisothiocyanate **2** with isobutyl amine was performed in THF for 1 h and the corresponding thiourea **5** was separated in 95% yield and characterized by ^1^H NMR analysis (See supporting information for ^1^H NMR spectra) (Scheme 2a). In continue, the reaction of freshly prepared thiourea **5** with nitroepoxide was carried out and the corresponding 2-iminothiazole **4e** was obtained in 90% isolated yield (Scheme 2b). According to these findings and literature reports^27,28,40,41^, a proposed mechanism for this reaction is depicted in Scheme 2c. Initially, reaction of amine with phenyl isothiocyanate furnish the corresponding *N,N’*-disubstituted thiourea **A**, which undergoes nucleophilic addition to nitroepoxide by sulfur atom to produce intermediate **B**. Elimination of nitrous acid from **B** affords the intermediate **C**. Then, intramolecular cyclization via the addition of aliphatic nitrogen to the carbonyl group in intermediate **C** provides the intermediate **D,** which undergoes dehydration reaction to afford the final structure **4**. The nitrogen of aliphatic amine is superior to the nitrogen of aromatic amine for cyclization reaction due to the higher electron density and nucleophilic character.

## Conclusion

In conclusion, we have developed an efficient catalyst-free one-pot three-component procedure for the synthesis of 2-imino-1,3-thiazoles in high to excellent yields. The reaction proceeds via the ring-opening of nitroepoxides with in situ prepared thiourea derivatives. By this protocol, it is possible to prepare diversities of 2-imino-1,3-thiazolines with various substitution pattern. The main advantages of this protocol include catalyst-free conditions, simple reaction conditions, high to excellent yields and convenient operations without extra purification.

## Supplementary Information

Supplementary Information.

## Data Availability

All data generated or analyzed during this study are included in this published article [and its Supplementary Information file].
