# Supplementary material for: Catalyst free one pot three components synthesis of 2-iminothiazoles from nitroepoxides and thiourea
Source: Sci Rep. 2023 Feb 22;13:3079. doi: 10.1038/s41598-023-30243-5 (PMC9947138; doi:10.1038/s41598-023-30243-5)
Supplement: Supplementary file 1 — Supplementary Information. [file 41598_2023_30243_MOESM1_ESM.docx]

**Catalyst free one pot three components synthesis of 2-iminothiazoles from nitroepoxides and thiourea**

Elham Badali,^a^ Azim Ziyaei Halimehjani,*^,a,b^ Azizollah Habibi^a^

*^a^ Faculty of Chemistry, Kharazmi University, 49 Mofateh St., 15719-14911, Tehran, Iran,
^b^ Department of Chemistry, Sharif University of Technology, P.O.Box 11155-9516, Tehran, Iran.*

*E-mail:* [*ziyaei@khu.ac.ir*](mailto:ziyaei@khu.ac.ir) *and Azim.Ziyaei@sharif.edu*

**Contents** **Pages** Experimental section (general procedure and characterization

data for all compounds) 2-10

Copies of ^1^H and ^13^C NMR spectra for all products and thiourea **5** 11-30

Crystal data for compound **4p** 31-33

**Experimental section**

**General.** All chemicals and solvents were obtained from commercial sources and used as received. The ^1^H and ^13^C NMR spectra of products were recorded on a Bruker AMX 300 MHz spectrometers referenced to internal Me_4_Si at 0.00 ppm. Reaction monitoring was carried out by thin-layer chromatography using TLC silica gel 60 F254 plates. HRMS (High Resolution Mass Spectra) was measured on a THERMO SCIENTIFIC Advantage and a THERMO SCIENTIFIC Exactive instrument equipped with an APCI source in the positive-ion mode. Mass analysis was performed using Agilent Technology (HP); 5973 Network Mass Selective Detector equipped with an EI mode and ionization energy of 70eV. The temperatures of the electronic-impact ion source and MS quadrupole were 230 ᵒC. IR spectra were recorded on a Perkin-Elmer Spectrum RXI FT-IR Spectrometer. Nitroepoxides were prepared according to the literature procedures (Ibrahim, M. M., Grau, D., Hampel, F., & Tsogoeva, S. B. α-Nitro Epoxides in Organic Synthesis: Development of a One-Pot Organocatalytic Strategy for the Synthesis of Quinoxalines. Eur. J. Org. Chem. 1401-1405. https://doi.org/10.1002/ejoc.201301591 (2014); **New 1:** Nosood, Y. L., Ziyaei Halimehjani, A. & Gonzalez, F. V. Regioselective Opening of Nitroepoxides with Unsymmetrical Diamines, *J. Org. Chem*. 83, 1252–1258. <https://doi.org/10.1021/acs.joc.7b02795> (2018).

***General procedure for the synthesis of 4a-s***

In a test tube equipped with magnetic stirrer bar, an isothiocyanate (1.0 mmol, 1 equiv), an amine (1.2 equiv) and THF (4 mL) were mixed for 1h at room temperature. Progress of the reaction was monitored by TLC. After complete consumption of the isothiocyanate, the reaction temperature was decreased to 10-15 ᵒC and a nitroepoxide (1 equiv) was added and the mixture was stirred at 10-15 ᵒC for 6h. The solvent was removed under reduced pressure to afford yellow viscous oil. Purification was carried out by recrystallization in a minimum amount of MeOH or by column chromatography using silicagel and *n*-hexane/EtOAc (7/3) (for compounds **4f**, **4h**, and **4i**).

***N-(3-ethyl-4-methyl-5-phenylthiazol-2(3H)-ylidene)aniline (4a):*** Yellowish powder (89%); mp 96-97 ^o^C; ^1^H NMR (300 MHz, Chloroform-*d*) *δ* 7.36-7.34 (m, 7H), 7.25 – 6.94 (m, 3H), 4.10 (q, *J* = 6.8 Hz, 2H), 2.29 (s, 3H), 1.43 (t, *J* = 7.0 Hz, 3H) ppm; ^13^C NMR (75 MHz, CDCl_3_) *δ* 158.9, 151.0, 132.5, 130.0, 129.4, 128.8, 128.6, 127.2, 123.1, 121.8, 109.2, 39.9, 13.7, 12.5 ppm; EI-MS m/z (%): 294 (M^+^,100), 266, 190, 147, 105, 77; IR (KBr) *ν*_max_ (cm^-1^): 696, 705, 777, 827, 1057, 1204, 1328, 1395, 1585, 1620.

***3-benzyl-4-methyl-5-phenylthiazol-2(3H)-ylidene)aniline (4b):*** White powder (81%); mp 105-106 °C; ^1^H NMR (300 MHz, Chloroform-*d*) *δ* 7.44 – 7.28 (m, 12H), 7.24 – 6.99 (m, 3H), 5.36 (s, 2H), 2.19 (s, 3H) ppm; ^13^C NMR (75 MHz, CDCl_3_) *δ* 158.5, 153.1, 136.8, 132.1, 130.6, 129.4, 128.7, 128.7, 128.7, 128.6, 127.4, 126.7, 123.4, 121.8, 109.8, 48.0, 12.8 ppm; EI-MS m/z (%): 356 (M^+^), 265, 167 (100), 147, 115, 91, 65; IR (KBr) *ν*_max_ (cm^-1^): 694, 758, 923, 1073, 1186, 1329, 1392, 1488, 1576, 1614.

***N-(5-(2,4-dichlorophenyl)-3-ethyl-4-methylthiazol-2(3H)-ylidene)anilin (4c):*** White powder (89%); mp 99-100 ^o^C; ^1^H NMR (300 MHz, Chloroform-*d*) *δ* 7.47 (s, 1H), 7.34 (t, *J* = 7.8 Hz, 2H), 7.27 (d, *J* = 7.4 Hz, 2H), 7.11 (d, *J* = 7.5 Hz, 2H), 7.04 (t, *J* = 7.3 Hz, 1H), 4.03 (q, *J* = 7.0 Hz, 2H), 2.04 (s, 3H), 1.41 (t, *J* = 7.1 Hz, 3H) ppm; ; ^13^C NMR (75 MHz, CDCl_3_) *δ* 158.2, 151.6, 135.8, 134.6, 133.6, 132.3, 129.8, 129.7, 129.3, 127.1, 122.8, 121.5, 103.5, 39.7, 13.6, 12.7 ppm; HRMS (ESI) calcd for C_18_H_16_Cl_2_N_2_S (M+H)^+^ 363.0489, found: 363.0484; IR (KBr) *ν*_max_ (cm^-1^): 694, 759, 870, 1100, 1201, 1323, 1403, 1473, 1563, 1576, 1626.

***N-(3-isobutyl-4-methyl-5-(3-nitrophenyl)thiazol-2(3H)-ylidene)aniline (4d):*** Yellow powder (84%); mp 102-103 ^o^C; ^1^H NMR (300 MHz, Chloroform-*d*) *δ* 8.20 – 8.13 (m, 1H), 8.14 – 8.03 (m, 1H), 7.61 (d, *J* = 7.8 Hz, 1H), 7.52 (t, *J* = 7.9 Hz, 1H), 7.36 (t, *J* = 7.8 Hz, 2H), 7.11 – 7.03 (m, 3H), 3.81 (d, *J* = 7.5 Hz, 2H), 2.41 (m, 1H), 2.29 (s, 3H), 1.06 (d, *J* = 6.7 Hz, 6H) ppm; ^13^C NMR (75 MHz, DMSO-*d*_6_) *δ* 157.1, 151.0, 148.0, 134.6, 133.9, 133.8, 130.4, 129.5, 122.9, 122.4, 121.6, 121.1, 104.6, 51.1, 27.1, 19.9, 12.9 ppm; HRMS (ESI) calcd for C_20_H_21_N_3_O_2_S (M+H)^+^  368.1433, found: 368.1427; IR (KBr) *ν*_max_ (cm^-1^): 696, 733, 764, 894, 1063, 1201, 1346, 1396, 1523, 1611.

***N-(5-(4-chlorophenyl)-3-isobutyl-4-methylthiazol-2(3H)-ylidene)aniline (4e):*** Yellow powder (81%); mp 87-89 ^o^C; ^1^H NMR (300 MHz, Chloroform-*d*) *δ* 7.44 – 7.13 (m, 7H), 7.07 (d, *J* = 7.7 Hz, 2H), 3.78 (d, *J* = 7.6 Hz, 2H), 2.39 (m, 1H), 2.22 (s, 3H), 1.04 (d, *J* = 6.7 Hz, 6H) ppm; ^13^C NMR (75 MHz, DMSO-*d*_6_) *δ* 157.1, 151.6, 132.1, 131.5, 131.2, 130.0, 129.4, 128.7, 122.6, 121.0, 105.5, 51.0, 27.1, 19.9, 12.8 ppm; HRMS (ESI) calcd for C_20_H_21_ClN_2_S (M+H)^+^  357.1192, found: 357.1186; IR (KBr) *ν*_max_ (cm^-1^): 689, 766, 837, 1013, 1091, 1200, 1392, 1489, 1577.

***N-(5-(2,4-dichlorophenyl)-3-isobutyl-4-methylthiazol-2(3H)-ylidene)aniline (4f):*** Yellow viscous oil (72%); ^1^H NMR (300 MHz, DMSO-*d*_6_) *δ* 7.71 (s, 1H), 7.44 (s, 2H), 7.28 (t, *J* = 7.8 Hz, 2H), 6.96 (dd, *J* = 17.1, 7.5 Hz, 3H), 3.72 (d, *J* = 7.4 Hz, 2H), 2.29 (m, 1H), 1.98 (s, 3H), 0.94 (d, *J* = 6.7 Hz, 6H) ppm; ^13^C NMR (75 MHz, DMSO-*d*_6_) *δ* 157.4, 151.3, 135.0, 134.4, 133.9, 133.8, 129.6, 129.4, 129.3, 127.7, 122.6, 121.0, 101.9, 51.1, 26.8 19.8, 12.9 ppm; EI-MS m/z (%): 390 (M^+^), 334, 193, 173, 149, 111, 85, 71, 57 (100); IR (KBr) *ν*_max_ (cm^-1^) 695, 766, 1073, 1101, 1200, 1323, 1398, 1471, 1584, 1608.

***N-(5-(4-chlorophenyl)-3-ethyl-4-methylthiazol-2(3H)-ylidene)aniline (4g):*** White powder (89%); mp147-149 ^o^C; ^1^H NMR (300 MHz, DMSO-*d*_6_) *δ* 7.45 (d, *J* = 8.4 Hz, 2H), 7.36-7.32 (m, 4H), 7.09-7.05 (m, 3H), 4.02 (q, *J* = 6.7 Hz, 2H), 2.27 (s, 3H), 1.29 (t, *J* = 6.9 Hz, 3H) ppm; ^13^C NMR (75 MHz, DMSO-*d*_6_) *δ* (ppm): 157.9, 151.8, 132.7, 131.3, 130.3, 129.9, 129.4, 128.7, 122.9, 121.5, 107.2, 39.9, 13.6, 12.5 ppm; HRMS (ESI) calcd for C_18_H_17_ClN_2_S (M+H)^+^ 329.0879, found: 329.0874; IR (KBr) *ν*_max_ (cm^-1^): 698, 775, 836, 904, 1090, 1394, 1486, 1583, 1620.

***N-(3-butyl-5-(2,4-dichlorophenyl)-4-methylthiazol-2(3H)-ylidene)aniline (4h):***Yellow viscous oil (74%); ^1^H NMR (300 MHz, DMSO-*d*_6_) *δ* 7.68(s, 1H), 7.45-7.42 (m, 2H), 7.28 (t, *J* = 7.7 Hz, 2H), 6.99-6.49 (m, 3H), 3.89 (t, *J* = 7.4 Hz, 2H), 2.00 (s, 3H), 1.71-1.67 ( m, 2H), 1.38-1.33 (m, 2H), 0.93 (t, *J* = 7.30 Hz , 3H) ppm; ^13^C NMR (75 MHz, DMSO-*d_6_*) *δ* 157.0, 151.1, 134.9, 134.3, 133.8, 133.5, 129.6, 129.4, 129.3, 127.7, 122.6, 121.0, 102.0, 44.1, 29.7, 19.5, 13.7, 12.5 ppm; EI-MS m/z (%):335 (M-C_4_H_9_), 215, 189, 173, 149, 135, 119, 103, 91, 77, 57, 41 (100); IR (KBr) *ν*_max_ (cm^-1^): 696, 765, 1072, 1101, 1196, 1328, 1398, 1472, 1584, 1607.

***N-(3-benzyl-5-(4-chlorophenyl)-4-methylthiazol-2(3H)-ylidene)aniline (4i):*** Yellow viscous oil (73%); ^1^H NMR (300 MHz, Chloroform-*d*) *δ* 7.41 – 7.29 (m, 9H), 7.28 – 7.21 (m, 2H), 7.18 – 7.05 (m, 3H), 5.28 (s, 2H), 2.14 (s, 3H) ppm; ^13^C NMR (75 MHz, CDCl_3_) *δ* 158.5, 151.2, 137.0, 132.8, 131.0, 130.7, 129.8, 129.3, 128.7, 128.6, 127.3, 126.6, 123.0, 121.4, 107.5, 47.6, 12.8 ppm; EI-MS m/z (%):390 (M^+^), 299, 167 (100), 139, 91, 65, 43; IR (KBr) *ν*_max_ (cm^-1^): 696, 767, 838, 1091, 1391, 1489, 1575, 1610.

***N-(3-butyl-4-methyl-5-(3-nitrophenyl)thiazol-2(3H)-ylidene)aniline (4j):*** Yellow viscous oil (72%); ^1^H NMR (300 MHz, DMSO-*d*_6_) *δ* 8.06 (d, *J* = 8.1 Hz, 1H), 8.01 (s, 1H), 7.71 (d, *J* = 7.8 Hz, 1H), 7.62 (t, *J* = 7.9 Hz, 1H), 7.30 (t, *J* = 7.8 Hz, 2H), 6.98 (t, *J* = 9.3 Hz, 3H), 3.99 – 3.81 (m, 2H), 2.27 (s, 3H), 1.68 (dd, *J* = 15.9, 8.8 Hz, 2H), 1.37 (h, *J* = 7.3 Hz, 2H), 0.93 (t, *J* = 7.3 Hz, 3H) ppm; ^13^C NMR (75 MHz, DMSO-*d*_6_) *δ* 156.4, 151.0, 147.9, 134.4, 133.9, 133.4, 130.3, 129.4, 122.8, 122.2, 121.5, 121.1, 104.5, 44.1, 29.8, 19.6, 13.6, 12.4 ppm; EI-MS m/z (%):367 (M^+^), 311 (100), 275, 235, 189, 150, 119, 93, 76, 57, 43; IR (KBr) *ν*_max_ (cm^-1^): 697, 736, 767, 1196, 1347, 1528, 1580, 1616.

***N-(3-benzyl-4-methyl-5-(3-nitrophenyl)thiazol-2(3H)-ylidene)aniline (4k):*** Orange powder (81%); mp 97-98 ^o^C; ^1^H NMR (300 MHz, DMSO-*d*_6_) *δ* 8.11 (dd, *J* = 7.8, 1.7 Hz, 1H), 8.06 (t, *J* = 1.9 Hz, 1H), 7.79 (d, *J* = 8.0 Hz, 1H), 7.66 (t, *J* = 8.0 Hz, 1H), 7.42 – 7.28 (m, 7H), 7.07 – 6.94 (m, 3H), 5.28 (s, 2H), 2.21 (s, 3H) ppm; ^13^C NMR (75 MHz, CDCl_3_) *δ* 157.2, 150.5, 148.0, 137.0, 134.6, 133.6, 133.6, 130.4, 129.5, 128.7, 127.3, 126.6, 123.1, 122.4, 121.8, 121.1, 105.1, 47.2, 12.6 ppm; HRMS (ESI) calcd for C_23_H_19_N_3_O_2_S (M+H)^+^ 402.1276, found 402.1271; IR (KBr) *ν*_max_ (cm^-1^): 697, 736, 750, 1073, 1184, 1343, 1390, 1558, 1615.

***N-(3-ethyl-4-methyl-5-(p-tolyl)thiazol-2(3H)-ylidene)aniline (4l):*** White powder (85%); mp 145-147 ^o^C; ^1^H NMR (300 MHz, DMSO-*d*_6_) *δ* 7.34 (t, *J* = 7.8 Hz, 2H), 7.23 – 6.98 (m, 7H), 4.03 (q, *J* = 7.0 Hz, 2H), 2.36 (s, 3H), 2.25 (s, 3H), 1.40 (t, *J* = 7.1 Hz, 3H) ppm; ^13^C NMR (75 MHz, CDCl_3_) *δ* (ppm): 158.2, 152.0, 136.8, 129.8, 129.3, 129.2, 128.6, 122.6, 121.6, 109.9, 108.5, 39.7, 21.1, 13.6, 12.5 ppm; HRMS (ESI) Calcd for C_19_H_20_N_2_S (M+H)^+^ 309.1425, found 309.1420; IR (KBr) *ν*_max_ (cm^-1^): 698, 777, 823, 1058, 1200, 1329, 1395, 1583, 1621.

***N-(5-(4-chlorophenyl)-4-methyl-3-propylthiazol-2(3H)-ylidene)aniline (4m):*** White powder (84%); mp 100-101 ^o^C; ^1^H NMR (300 MHz, CDCl_3_) *δ* 7.36-7.27 (m, 7H), 7.22-7.04 (m, 2H), 3.91 (t, *J* = 7.7 Hz, 2H), 2.22 (s, 3H), 1.92-1.79 (m, 2H), 1.04 (t, *J* = 7.4 Hz, 3H) ppm; ^13^C NMR (75 MHz, CDCl_3_) *δ* 158.1, 151.9, 132.7, 131.3, 130.5, 129.94, 129.4, 128.7, 122.9, 121.5, 107.2, 46.3, 21.6, 12.7, 11.3 ppm; HRMS (ESI) calcd for C_19_H_19_ClN_2_S (M+H)^+^ 343.1036, found 343.1030; IR (KBr) *ν*_max_ (cm^-1^): 697, 779, 1090, 1198, 1326, 1486, 1583, 1620.

***N-(4-methyl-3-propyl-5-(p-tolyl)thiazol-2(3H)-ylidene)aniline (4n):*** White powder (85%), mp 109-110 ^o^C; ^1^H NMR (300 MHz, Chloroform-*d*) *δ* 7.35 (t, *J* = 7.8 Hz, 2H), 7.23 – 7.13 (m, 4H), 7.10 (d, *J* = 8.1 Hz, 2H), 7.05 (t, *J* = 7.3 Hz, 1H), 3.92 (t, *J* = 7.69 Hz, 2H), 2.37 (s, 3H), 2.25 (s, 3H), 1.88-1.86 (m, 2H), 1.06 (t, *J* = 7.4 Hz, 3H) ppm; ^13^C NMR (75 MHz, CDCl_3_) *δ* 158.5, 152.1, 136.8, 129.8, 129.5, 129.3, 129.2, 128.6, 122.6, 121.6, 108.4, 46.2, 21.6, 21.1, 12.7, 11.3 ppm; EI-MS m/z (%): 322 (M^+^), 307, 280 (100), 230, 204, 150, 135, 115, 91, 69, 41; IR (KBr) *ν*_max_ (cm^-1^): 697, 778, 820, 1197, 1326, 1486, 1583, 1621.

***4-chloro-N-(5-(4-chlorophenyl)-3-ethyl-4-methylthiazol-2(3H)-ylidene)aniline (4o):*** White powder (88%); mp 120-121 ^o^C; ^1^H NMR (300 MHz, CDCl_3_) *δ* 7.34-7.27 (m, 4H), 7.22 (d, *J* = 8.6 Hz, 2H), 7.03 (d, *J* = 8.6 Hz, 2H), 4.01 (q, *J* = 7.1 Hz, 2H), 2.23 (s, 3H), 1.38 (t, *J* = 7.1 Hz, 3H) ppm; ^13^C NMR (75 MHz, CDCl_3_) *δ* 158.5, 150.3, 132.9, 131.1, 130.4, 129.9, 129.3, 128.8, 127.6, 122.9, 107.4, 39.7, 13.6, 12.5 ppm; HRMS (ESI) calcd for C_18_H_16_Cl_2_N_2_S (M+H)^+^ 363.0489, found 363.0484; IR (KBr) *ν*_max_ (cm^-1^): 694, 759, 870, 1100, 1201, 1323, 1403, 1473, 1563, 1576, 1626.

***4-chloro-N-(4-methyl-3-propyl-5-(p-tolyl)thiazol-2(3H)-ylidene)aniline (4p):*** White powder (86%); mp 78-79 ^o^C; ^1^H NMR (300 MHz, CDCl_3_) *δ* 7.29-7.27 (d, *J* = 8.6 Hz, 2H), 7.21-7.15 (m, 4H), 7.05-7.00 (m, 2H), 3.90 (t, *J* = 6.7 Hz, 2H), 2.37 (s, 3H), 2.25 (s, 3H), 1.91-1.79 (m, 2H), 1.04 (t, *J* = 7.4 Hz, 3H) ppm; ^13^C NMR (75 MHz, CDCl_3_) *δ* 158.8, 150.6, 137.0, 129.6, 129.6, 129.3, 128.7, 127.4, 123.0, 109.9, 108.7, 46.3, 21.6, 21.1, 12.7, 11.3 ppm; HRMS (ESI) calcd for C_20_H_21_ClN_2_S 357.1192, found 357.1187; IR (KBr) *ν*_max_ (cm^-1^): 813, 848, 1089, 1197, 1326, 1392, 1482, 1580, 15.99, 1617.

***4-chloro-N-(5-(2,4-dichlorophenyl)-3-ethyl-4-methylthiazol-2(3H)-ylidene)aniline (4q):*** White powder (88%); mp 97-98 ^o^C; ^1^H NMR (300 MHz, Chloroform-*d*) *δ* 7.47 (t, *J* = 1.0 Hz, 1H), 7.28 (d, *J* = 2.8 Hz, 1H), 7.27 – 7.22 (m, 3H), 7.08 – 6.99 (m, 2H), 4.01 (q, *J* = 7.1 Hz, 2H), 2.03 (s, 3H), 1.39 (t, *J* = 7.1 Hz, 3H) ppm; ^13^C NMR (75 MHz, CDCl_3_) *δ* 158.5, 150.1, 135.8, 134.7, 133.6, 132.4, 129.8, 129.6, 129.3, 127.5, 127.2, 122.8, 103.7, 39.7, 13.5, 12.7 ppm; HRMS (ESI) calcd for C_18_H_15_Cl_3_N_2_S (M+H)^+^ 399.0070, found 399.0065; IR (KBr) *ν*_max_ (cm^-1^): 694, 759, 870, 1100, 1201, 1323, 1403, 1473, 1563, 1576, 1617.

***N-(5-(4-chlorophenyl)-3-ethyl-4-methylthiazol-2(3H)-ylidene)-4-nitroaniline (4r):*** Orange powder (93%); mp 142-143 ^o^C; ^1^H NMR (300 MHz, CDCl_3_) *δ* 8.22 (d, *J* = 8.8 Hz, 2H), 7.39(d, J = 8.4 Hz, 2H), 7.39-7.19 (m, 5H), 4.86 (q, *J* = 6.90 Hz, 2H), 2.29 (s, 3H), 1.41 (t, *J* = 6.9 Hz, 3H) ppm; ^13^C NMR (75 MHz, CDCl_3_) *δ* 158.5, 157.5, 142.6, 133.9, 130.9, 130.1, 129.0, 125.5, 123.7, 121.4, 109.1, 40.2, 13.6, 12.5 ppm; HRMS (ESI) calcd for C_18_H_16_ClN_3_O_2_S (M+H)^+^ 374.0730, found 374.0725; IR (KBr) *ν*_max_ (cm^-1^): 856, 1109, 1310, 1392, 1489, 1526, 1583, 1612.

***N-(4-methyl-3-propyl-5-(p-tolyl)thiazol-2(3H)-ylidene)-4-nitroaniline (4s):*** Red powder (90%); mp 99 ^o^C; ^1^H NMR (300 MHz, CDCl_3_) *δ* 8.22 (d, *J* = 8.8 Hz, 2H), 7.28-7.21 (m, 6H), 3.96 (t, *J* = 7.6 Hz, 2H), 2.38 (s, 3H), 2.28 (s, 3H), 1.92-1.79 (m, 2H), 1.05 (t, *J* = 7.4 Hz, 3H) ppm; ^13^C NMR (75 MHz, CDCl_3_) *δ* 159.1, 157.5, 141.9, 137.63, 129.8, 129.4, 128.9, 128.8, 125.4, 121.4, 110.1, 46.7, 21.7, 21.1, 12.7, 11.3 ppm; HRMS (ESI) calcd for C_20_H_21_N_3_O_2_S (M+H)^+^ 368.1433, found 368.1427; IR (KBr) *ν*_max_ (cm^-1^): 856, 1109, 1318, 1537, 1609.

**Copies of ^1^H and ^13^C NMR spectra for all products and thiourea 5**

**Figure 1.** ^1^H NMR spectrum for **4a**

**Figure 2.** ^13^C NMR spectrum for **4a**

**Figure 3.** ^1^H NMR spectrum for **4b**

**Figure 4.** ^13^C NMR spectrum for **4b**

**Figure 5.** ^1^H NMR spectrum for **4c**

**Figure 6.** ^13^C NMR spectrum for **4c**

**Figure 7.** ^1^H NMR spectrum for **4d**

**Figure 8.** ^13^C NMR spectrum for **4d**

**Figure 9.** ^1^H NMR spectrum for **4e**

**Figure 10.** ^13^C NMR spectrum for **4e**

**Figure 11.** ^1^H NMR spectrum for **4f**

**Figure 12.** ^13^C NMR spectrum for **4f**

**Figure 13.** ^1^H NMR spectrum for **4g**

**Figure 14.** ^13^C NMR spectrum for **4g**

**Figure 15.** ^1^H NMR spectrum for **4h**

**Figure 16.** ^13^C NMR spectrum for **4h**

**Figure 17.** ^1^H NMR spectrum for **4i**

**Figure 18.** ^13^C NMR spectrum for **4i**

**Figure 19.** ^1^H NMR spectrum for **4j**

**Figure 20.** ^13^C NMR spectrum for **4j**

**Figure 21.** ^1^H NMR spectrum for **4k**

**Figure 22.** ^13^C NMR spectrum for **4k**

**Figure 23.** ^1^H NMR spectrum for **4l**

**Figure 24.** ^13^C NMR spectrum for **4l**

**Figure 25.** ^1^H NMR spectrum for **4m**

**Figure 26.** ^13^C NMR spectrum for **4m**

**Figure 27.** ^1^H NMR spectrum for **4n**

**Figure 28.** ^13^C NMR spectrum for **4n**

**Figure 29.** ^1^H NMR spectrum for **4o**

**Figure 30.** ^13^C NMR spectrum for **4o**

**Figure 31.** ^1^H NMR spectrum for **4p**

**Figure 32.** ^13^C NMR spectrum for **4p**

**Figure 33.** ^1^H NMR spectrum for **4q**

**Figure 34.** ^13^C NMR spectrum for **4q**

**Figure 35.** ^1^H NMR spectrum for **4r**

**Figure 36.** ^13^C NMR spectrum for **4r**

**Figure 37.** ^1^H NMR spectrum for **4s**

**Figure 38.** ^13^C NMR spectrum for **4s**

**Figure 39.** ^1^H NMR spectrum of thiourea **5**

**X-Ray crystallography**

Suitable crystals for X-ray crystallography were grown in ethanol by slow evaporation of solvent at room temperature.

| **X-ray Crystallography**  Table 1. Crystal data of compound **4p** (CCDC- 217830 ) | |
| --- | --- |
| Empirical formula | C_20_H_21_ClN_2_S |
| Formula weight | 356.90 |
| Temperature | 290 |
| Wavelength | 0.71073 |
| Crystal system | orthorhombic |
| Space group | *pbca* |
| Unit cell dimensions | a = 13.808(3) Å *α* = 90 °  b = 14.504(3) Å *β* = 90 °  c = 18.506(4) Å *γ* = 90 ° |
| Volume | 3706.2 (13) Å^3^ |
| Z | 8 |
| Calculated density | 1.279 Mg/m^3^ |
| Absorption coefficient | 0.322 mm^-1^ |
| F(000) | 1504 |
| Crystal size | 0.300 x 0.200 x 0.100 mm^3^ |
| Theta range for data collection | 2.3 to 25.9 ° |
| Index ranges | -16<=h<=16, -17<=k<=17, -22<=l<=22 |
| Reflections collected | 24481 |
| Independent reflections | 3600 [R(int) = 0.0471] |
| Data / restraints / parameters | 3600/0/220 |
| Goodness-of-fit on F^2^ | 1.122 |
| Final R indices [I>2σ(l)] | R1 = 0.0449, ωR2 = 0.1075 |
| R indices (all data) | R1 = 0.0471, ωR2 = 0.1090 |
| Largest diff. peak and hole | 0.174 and -0.220 e×Å3 |


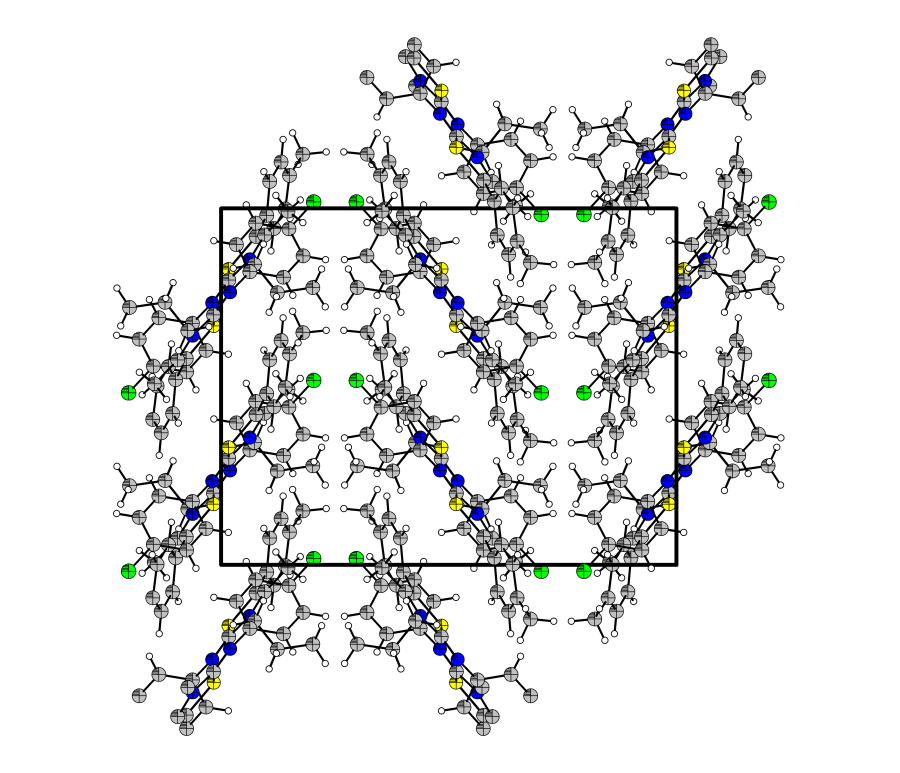


**Figure 40.** Crystal structure for **4p** (Pack-a). The figure was drawn by DIAMOND (https://www.crystalimpact.com/diamond/).^1^
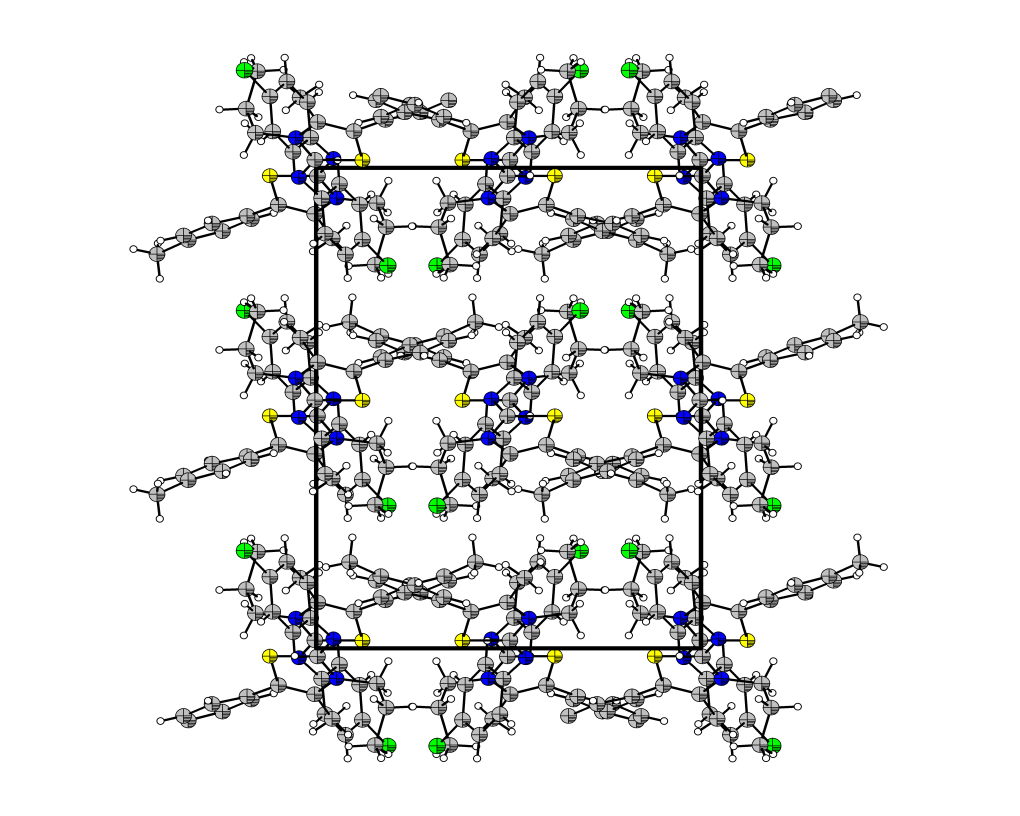


**Figure 41.** Crystal structure for **4p** (Pack-b). The figure was drawn by DIAMOND (https://www.crystalimpact.com/diamond/).^1^


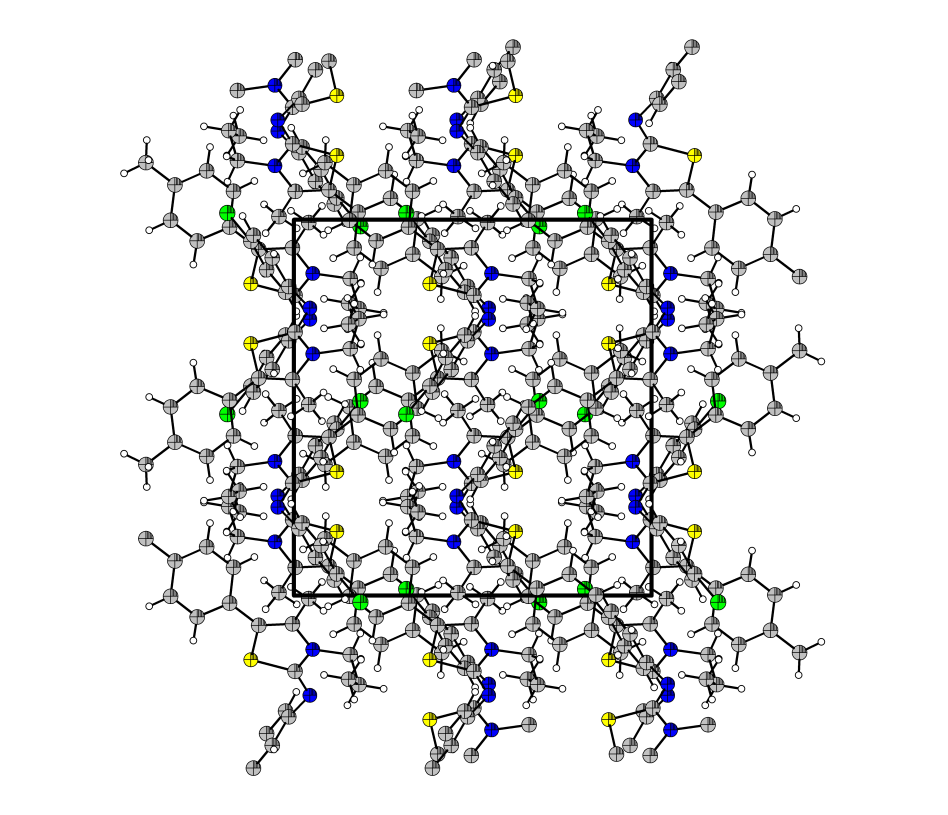


**Figure 42.** Crystal structure for **4p** (Pack-c). The figure was drawn by DIAMOND (https://www.crystalimpact.com/diamond/).^1^

1. Pennington, W. T., DIAMOND– Visual Crystal Structure Information System, *J. Appl. Crystallogr*. 32, 1028-1029. <https://doi.org/10.1107/S0021889899011486> (1999)
